# Local ASIC3 modulates pain and disease progression in a rat model of osteoarthritis

**DOI:** 10.1186/1423-0127-19-77

**Published:** 2012-08-21

**Authors:** Masashi Izumi, Masahiko Ikeuchi, Qinghui Ji, Toshikazu Tani

**Affiliations:** 1 Department of Orthopaedic Surgery, Kochi University, Oko-cho Kohasu, Nankoku, 783-8505, Japan

**Keywords:** Osteoarthritis, Acid sensing ion channel (ASIC), Pain, APETx2, Joint, Inflammation

## Abstract

**Background:**

Recent data have suggested a relationship between acute arthritic pain and acid sensing ion channel 3 (ASIC3) on primary afferent fibers innervating joints. The purpose of this study was to clarify the role of ASIC3 in a rat model of osteoarthritis (OA) which is considered a degenerative rather than an inflammatory disease.

**Methods:**

We induced OA *via* intra-articular mono-iodoacetate (MIA) injection, and evaluated pain-related behaviors including weight bearing measured with an incapacitance tester and paw withdrawal threshold in a von Frey hair test, histology of affected knee joint, and immunohistochemistry of knee joint afferents. We also assessed the effect of ASIC3 selective peptide blocker (APETx2) on pain behavior, disease progression, and ASIC3 expression in knee joint afferents.

**Results:**

OA rats showed not only weight-bearing pain but also mechanical hyperalgesia outside the knee joint (secondary hyperalgesia). ASIC3 expression in knee joint afferents was significantly upregulated approximately twofold at Day 14. Continuous intra-articular injections of APETx2 inhibited weight distribution asymmetry and secondary hyperalgesia by attenuating ASIC3 upregulation in knee joint afferents. Histology of ipsilateral knee joint showed APETx2 worked chondroprotectively if administered in the early, but not late phase.

**Conclusions:**

Local ASIC3 immunoreactive nerve is strongly associated with weight-bearing pain and secondary hyperalgesia in MIA-induced OA model. APETx2 inhibited ASIC3 upregulation in knee joint afferents regardless of the time-point of administration. Furthermore, early administration of APETx2 prevented cartilage damage. APETx2 is a novel, promising drug for OA by relieving pain and inhibiting disease progression.

## Background

Osteoarthritis (OA) is one of the most common joint diseases characterized by degeneration of articular cartilage, osteophyte formation, subchondral bone sclerosis, and secondary synovitis. Although a major symptom of OA is chronic joint pain which has a significant effect on patients’ quality of life, the pain mechanisms remain largely unknown. One of the reasons is that the pathophysiology of joint pain associated with degeneration is more complicated than acute inflammatory joint pain. Although anti-inflammatory drugs are still the class of medication most commonly used in OA treatment, they are insufficient to relieve pain.

Acid sensing ion channels (ASICs) are sodium-selective ion channels activated by low extracellular pH, and belong to the degenerin/epithelial Na^+^ channel superfamily [1]. Among ASICs, ASIC3 is the most sensitive to such a pH change [2,3], abundantly expressed in dorsal root ganglia (DRG) [4], and strongly correlated with pain [5-12]. In recent years, there has been considerable evidence suggesting that ASIC3 plays a significant role in joint inflammatory pain [13-15]. Our previous reports showed that secondary hyperalgesia following carrageenan-induced arthritis (response to von-Frey filaments applied to the paw) does not develop in ASIC3 knockout mice while primary mechanical hyperalgesia (response to tweezer applied to the inflamed knee joint) develops similarly between knockout and wildtype mice. We concluded ASIC3 is critical for the development of secondary hyperalgesia [16]. In addition, ASIC3 immunoreactive peripheral nerves were upregulated in inflamed synovium of the knee joint and dosal root ganglia (DRG) along with calcitonin gene-related peptide (CGRP) [16,17]. However, carrageenan-induced arthritis is an experimental model of acute joint inflammation. There has been no evidence yet for the relationship between ASIC3 and OA, which is considered a degenerative rather than an inflammatory disease.

The purpose of this study was, therefore, to test the role of ASIC3 in a rat knee OA model compared with naïve rats. Specifically, we examined a pain-related animal behavior test, histological change of knee joint, and expression of ASIC3 in knee joint afferents. We also examined the effect of intra-articular injection of ASIC3-selective peptide blocker, APETx2 [18].

## Methods

### Induction of OA

Male Sprague–Dawley rats (8 weeks old, weight 230-270 g) were used. All experiments were approved by the Animal Care and Use Committee of Kochi University. After acclimation to the facility, rats were anesthetized *via* intraperitoneal injection of sodium pentobarbital. To induce OA, 3 mg of mono-iodoacetate (MIA) dissolved in 50 μl saline were injected into the left knee using a 27-gauge needle [19-23].

Intra-articular injection of MIA inhibits glyceraldehyde-3-phosphate dehydrogenase activity in chondrocytes, resulting in disruption of glycolysis and eventual death of chondrocytes [24-27]. This process usually accompanies initial inflammatory response, histologically known as expansion of synovial membrane, infiltration of macrophages, neutrophils, and lymphocytes. In the later phase, however, degenerative change predominantly exists without histological inflammation. Therefore, pathophysiology of joint pain in this model is considered completely different from the acute inflammatory arthritis model.

Compared to other experimental models, the MIA-induced OA model is highly reproducible and mimics OA pain in humans [28]. Histological changes include cartilage degradation [27,29], subchondral bone changes [22,30], synovial inflammation [19], and osteophyte formation [19,27,29]. Although prominent inflammation generally resolves in the early phase [19], sustained elevation of proinflammatory cytokines is observed even after the disappearance of inflammatory infiltrates [31,32]. Pain behaviors include weight bearing pain, tactile allodynia, and mechanical hyperalgesia [33]. Therefore, many authors currently use this model as an established OA model [19-23,27-36]. In addition, clinical studies suggest the existence of neuropathic component in OA pain [37,38], and several papers showed MIA injection into rat knee joint evoked not only inflammation and degenerative change, but also possible localized neuropathic component involving joint afferent [32,35,39]. Therefore, this OA model is suitable for our aim to examine the role of ASIC3 on joint tissues and joint afferents.

### Animal behavior assessment

For animal behavior test, 10 rats in each group were employed. Pain-related behaviors were assessed using a hind paw limb weight-bearing apparatus (Linton incapacitance tester, Norfolk, UK) and von Frey filaments at pre- and post-MIA injection. Animals were acclimated for 30 min before each assessment. A comparison between OA-model and naïve rats was continued for 28 days after MIA injection. The incapacitance tester automatically showed the difference in weight bearing between the ipsilateral affected limb and the contralateral control limb. Measurement was performed five times in each rat, and the average of middle three values was calculated. Percent weight distribution of left (ipsilateral) hind paw was calculated by the following formula [22]:

(1) $$\%\;\text{weight distribution of left hind paw}=\text{left weight}/\left(\text{left weight}+\text{right weight}\right)\times 100$$

The frequency of paw withdrawal reflex to 10 g von Frey filaments was counted from ten trials. The value of % weight distribution or paw withdrawal reflex was represented as the mean of all measurements.

### Retrograde labeling of knee joint afferents

At Day 8 after MIA injection, animals were deeply anesthetized with intraperitoneal sodium pentobarbital injection. After shaving, a 5 mm long skin incision was made at the left knee joint and 0.1 mg Fast Blue (FB) (Polysciences, Inc. Warrington, PA) diluted in 10 μl of saline was injected into the joint cavity for retrograde labeling. A careful check was made to ensure that no FB had leaked into the surrounding tissues and the wound was closed with 5–0 nylon. FB containing neurons were identified *in vitro* by blue fluorescence on brief exposure of the cells to ultraviolet light [40].

### Immunohistochemistry of DRGs

At Day14 after MIA injection (6 days after FB injection), animals were euthanized with an overdose of sodium pentobarbital (150 mg/kg, i.p.), and the ipsilateral lumbar DRGs (L3-L5) were obtained. The DRGs were placed in 4% paraformaldehyde and 30% sucrose overnight, embedded in OCT compound (Sakura Finetek, Torrace, CA, USA) and frozen in −80°C until sectioning. Ten-micrometer frozen sections were then cut using a cryostat.

The sections were blocked in 3% normal goat serum for 1 h, then incubated in primary antibody of ASIC3 (Neuromics; Edina, MN, GP 14015, 1:500) overnight in a humid chamber. The next day, the sections were incubated in the secondary antibody (Vector; Burlingame, CA, FI-7000, 1:500, FITC tagged) for 2 h. All antisera used were diluted in PBS containing 1% normal goat serum and 0.05% Triton X-100. Before, between, and after each incubation step, the sections were washed 3 times for 5 min in PBS. Finally, all sections were mounted with Vectashield (Vector, Burlingame, CA).

Sections were viewed with a Nikon Eclipse 80i microscope (Nikon, Tokyo, Japan). Representative photos of DRGs were taken using DS-Ri1 CCD camera (Nikon, Tokyo, Japan). FB-labeled neurons from every fifth section were counted to eliminate the possibility of double counting. More than 100 FB-labeled neurons were analyzed from 4 rats in each group. ASIC3 expression was counted in FB-labeled neurons manually and quantified as the percent of total FB-labeled neurons.

### Histological evaluation of knee joint

The knees were placed in 10% formalin, decalcified by formic acid for 3 days, and embedded in paraffin. Five-micrometer sections were cut and stained with 0.1% safranin O. Histopathologic classification on the severity of the OA lesion was graded on a scale of 0–13 (from 0 (worst) to 13 (best)), using the modified Mankin scoring system [41].

### Administration of selective ASIC3 blocker (APETx2)

APETx2 is a 42-amino acid peptide isolated from venom of sea anemone, which is a highly selective blocker for ASIC3 [18]. The concentration of APETx2 (Alomone labs, RTA-100, Jerusalem, Israel) was determined as 2.5 μg/kg, according to a previous report [42]. The procedures of anesthetization and intra-articular injection were the same as MIA administration. Preliminarily, rats were given a single injection of APETx2 at Days 1, 7 and 14 after MIA injection. The results showed that each injection had a temporary effect on paw withdrawal reflex, but the differences were not statistically significant (data not shown). Therefore, repeated daily injections were performed from Days 1 to 7 (early-phase group) or from Days 7 to 13 (late-phase group). As a control, repeated daily injections of saline were also performed (OA-saline group). Pain-related behaviors were assessed by weight distribution and von Frey test at Days 0, 1, 3, 7, 8, and 14 in each group.

### Statistical analysis

Statistical analysis was carried out using JMP, Version 9 (SAS Ins. Cary, NC). Two-way analysis of variance (ANOVA) followed by Tukey’s test was used to compare animal behavior tests. A chi-square test was used for evaluating the percentage of ASIC3 immunoreactive neurons in DRGs. Kruskal-Wallis test with Steel-Dwass test was used for comparing histological evaluations of knee joints. A *p*-value of <0.05 was considered statistically significant.

## Results

### OA vs Naïve

**Figure 1:**
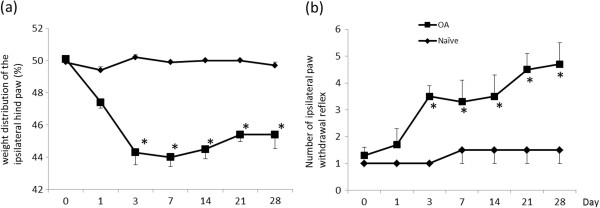
**The effect of OA induction on behavior tests.** (**a**) Percentage of weight distribution of the ipsilateral hind paw using an incapacitance tester, (**b**) Number of ipsilateral paw withdrawal reflex to 10 g von Frey filament. Pain-related behavior, i.e. asymmetric weight distribution and mechanical hyperalgesia of the paw were observed from Day 3 to Day 28 in the OA-model group. * *p* < 0.05 compared to Naïve-model rats.

**Figure 2:**
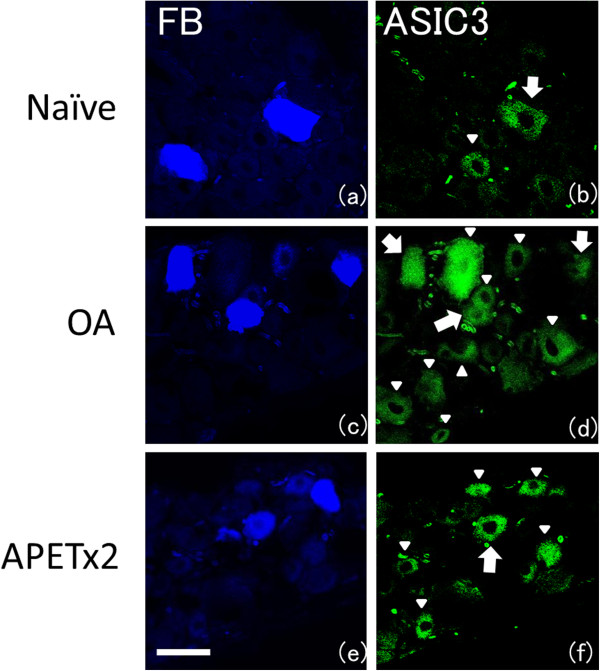
**Fast Blue labeling and immunohistochemistry staining for ASIC3 : (a-b) Naïve- model, (c-d) OA-model, (e-f) APETx2 administration to OA-model in early phase.** Photos in each row are the same DRG. In (**b**),(**d**),(**f**), large arrows indicate Fast Blue labeled, ASIC3 immunoreactive (ASIC3-ir) DRG cells, while ASIC3-ir cells that were not labeled by Fast Blue are indicated by small arrowheads. More than 100 FB-labeled neurons were analyzed from 4 rats in each group. The percentage of ASIC3-ir knee joint afferents was 18 ± 3% (mean ± SD) in naïve models, 46 ± 4% in OA-models (*p* = 0.003), and 20 ± 5% in the early-phase APETx2 group (*p* = 0.006), respectively. Scale bar: 50 μm.

**Figure 3:**
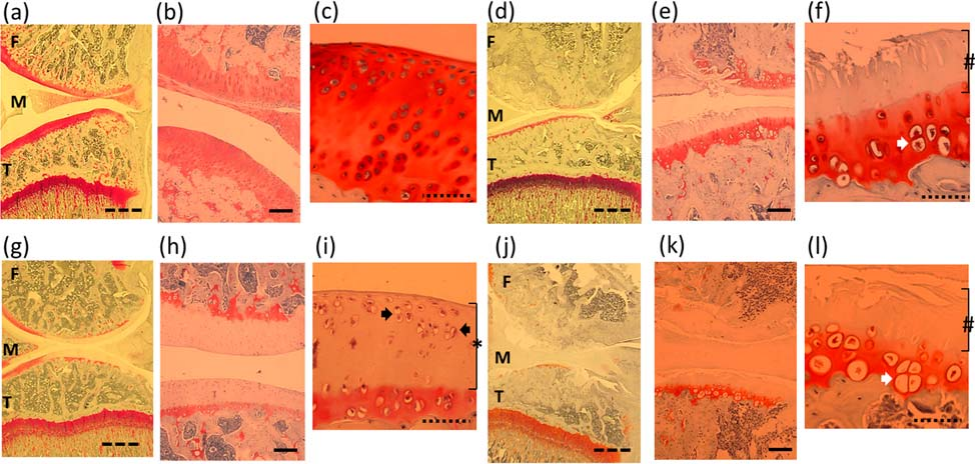
**Histology of knee joints with Safranin O staining at Day14.** Three different magnifications (×1.25, ×10, ×20) were shown in each group. (**a-c**) Naïve model: Full thickness of cartilage. Rich chondrocytes with proteoglycan (red staining by safranin O) (**d-f**) OA model: Severe damage of cartilage surface with loss of chondrocytes in superficial and middle layer (#), hypertrophied chondrocytes in deep zone (white arrow) were observed in (**f**) . Increased thickening of subchondral bone subjacent to the area of severe cartilage lesion was also observed in (**d,e**). (**g-i**) APETx2 administration in early phase: Chondrocytes were well observed in superficial and middle layer (black arrow). Although proteoglycan loss, cartilage surface kept smooth and no apparent thinning (*) in (**i**). (**j-l**) APETx2 in late phase: same findings as OA. Apparent chondroprotective effect was not seen (#) and hypertrophied chondrocytes in deep zone were also observed (white arrow) in (**l**). F: femur, M: meniscus, T: tibia, Scale bar: 1 mm, 100 μm 50 μm.

Figure1 shows the effect of OA induction on behavior tests. Pain-related behavior, i.e. asymmetric weight distribution and mechanical hyperalgesia of the paw was observed from Day 3 to Day 28 in the OA group (a,b). Figure2 shows representative photos of DRGs with immunohistochemical staining. The percentage of ASIC3 immunoreactive knee joint afferents was 18 ± 3% (mean ± SD) in naïve models, 46 ± 4% in OA-models (*p* = 0.003), respectively. In the OA model, histopathological findings showed loss of chondrocytes and cartilage matrix in superficial and middle zone, and hypertrophic change of chondrocytes in deep zone. In addition, OA related changes such as cartilage thinning, surface irregularity, and thickening of the subchondral bone were also observed. (Figure3 d-f).

### Effects of APETx2

**Figure 4:**
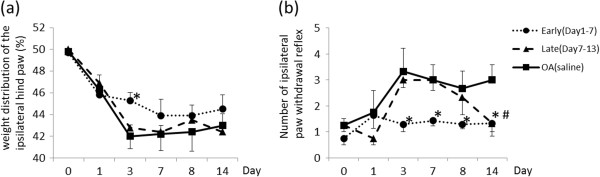
**The effect of intra-articular injection of APETx2 on behavior tests.** (**a**)weight distribution, (**b**) paw withdrawal reflex (same manner as Figure1). Weight distribution was changed significantly at Day3 in early APETx2 administration group. Frequency of paw withdrawal reflex i.e. secondary hyperalgesia reduced with APETx2 injection. The inhibitory effects on secondary hyperalgesia in both early- and late-phase groups were observed at Day14. * *p* < 0.05 (Early vs OA), # *p* < 0.05 (Late vs OA).

Weight distribution asymmetry was affected by continuous daily APETx2 injections in the early-phase group, significantly at Day3 (Figure4a). APETx2 injections resulted in a significant decrease in the frequency of paw withdrawal reflex, i.e. reduced secondary hyperalgesia, until Day 14. The inhibitory effects on secondary hyperalgesia were similar in the early and late phase groups at Day 14 (Figure4b).

**Figure 5:**
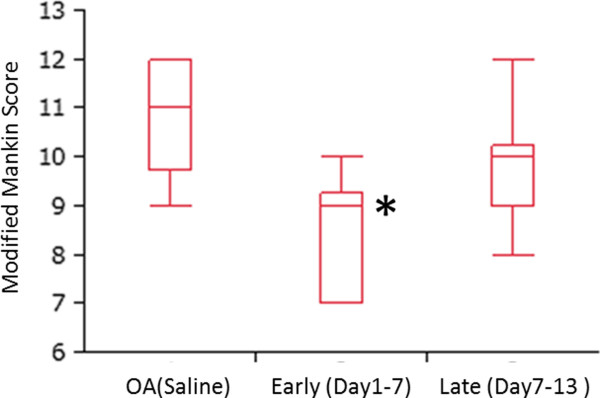
**Histological evaluation of knee joint using Modified Mankin Score.** a scale of 0–13 (from 0 (worst) to 13 (best)). Intra-articular injection of APETx2 in early phase prevented OA progression, including breakdown of articular surface and hypocellularity. * *p* < 0.05 compared to OA.

Histology of knee joints showed that early administration of APETx2 resulted in reducing OA severity (Figure3g-i). However, late administration had no significant effect on knee histology (Figure3j-l). Modified Mankin score (from 0 (worst) to 13 (best)) was 9 [7–9.3] (median [range]) in the early-phase group, 10 [9–10.3] in the late-phase group, and 11 [9.8-12] in the OA-saline group (Figure5). The modified Mankin score consists of 3 subgroups, structure (0–6), cellular abnormalities (0–3), and matrix staining (0–4) [41]. Although matrix staining with Safranin O showed limited improvement by APETx2 injection, however, breakdown of articular surface and hypocellularity were apparently prevented, which resulted in a significant difference of Mankin score between early- and late- groups.

Immunohistochemical staining of DRG showed that the percentage of ASIC3 immunoreactive neurons in FB-labeled neurons was 46 ± 4% in OA, 20 ± 5% in the early-phase group (*p* = 0.006), and 19 ± 3% in the late-phase group (*p* = 0.003), respectively. ASIC3 expression in knee joint afferents was upregulated more than twofold at Day 14 after inducing OA. Interestingly, continuous administration of APETx2, not only the early- but also the late-phase inhibited ASIC3 upregulation.

## Discussion

The present study provides new insights into the pain mechanisms and disease progression of OA associated with ASIC3. Compared to naïve models, OA-model rats exhibited not only weight-bearing pain but also secondary hyperalgesia. Histology of knee joints showed OA changes consistent with previous reports [19,27,29,30,33]. ASIC3 expression in knee joint afferents was significantly upregulated by inducing OA. Intra-articular injection of APETx2, a specific blocker of ASIC3, had an inhibitory effect on weight distribution asymmetry and secondary hyperalgesia by attenuating ASIC3 upregulation in knee joint afferents. In addition, APETx2 showed chondroprotective effect on OA rats in early- but not late-phase administration.

This is the first report to describe the relationship between OA and ASIC3. MIA-induced OA model has initial inflammation and subsequent degeneration phases. Initial inflammation decreases local pH and upregulates ASIC3 expression in knee joint afferents [17]. Although the histology showed little inflammation at Day 14, ASIC3 expression was possibly maintained by sustained elevation of proinflammatory cytokines [31,32]. Furthermore, recent publications have shown that nerve growth factor (NGF) in local tissues increased in the degeneration phase of MIA-induced OA [31,32]. NGF has also been found to upregulate ASIC3 expression in addition to sensitize nociceptors [43,44].

ASIC3 in knee joint afferents was not upregulated by APETx2 regardless of the time-point of administration. There are two possible mechanisms. Firstly, neurogenic inflammation *via* the activation of peptidergic neurons was possibly inhibited. When neuropeptides such as calcitonin gene–related peptide (CGRP) and substance P are released from primary afferent fibers peripherally, they act on target cells such as mast cells, immune cells, and vascular smooth muscle producing local inflammation [45]. Then, the target cells release proinflammatory mediators including NGF, serotonin and bradykinin, which are major inducers of ASIC3 upregulation [43]. Previous studies showed that ASIC3 and CGRP were co-expressed on joint and muscle afferents [17,46]. Therefore, APETx2 possibly inhibit neuropeptides release from nerve terminals. Secondly, APETx2 could attenuate the activity-dependent gene regulation in nociceptors by inhibiting ASIC3 and Nav1.8 [47]. The activity of nociceptor endings depends on the gating properties of ion channels in the membrane. During the process of sensitization, the expression of ion channels is regulated such that more molecules are available for stimulation [48,49]. APETx2 could attenuate a depolarizing sensor potential, which possibly inhibited nociceptor activation and subsequent upregulation of ASIC3.

In terms of pain-related behavior tests, APETx2 significantly inhibited secondary hyperalgesia assessed by von Frey filaments, which was consistent with our previous study of acute arthritic models [16]. However, a significant difference in weight distribution was observed only at Day 3 in the early-phase injection. Compared with von Frey test, evaluation of weight distribution is a less sensitive technique, and the apparatus may be unable to detect minor differences. Meanwhile, considering the therapeutic potential in OA, it is rather beneficial that APETx2 did not inhibit weight distribution asymmetry completely, because it might be necessary to prevent further joint damage in OA.

Surprisingly, APETx2 injection into the knee joint prevented cartilage damage, only in the early phase. Previous studies showed the relationship between joint acidosis and cartilage damage. *In vitro*, extracellular low pH not only inhibited matrix synthesis by chondrocytes [50,51], but also induced chondrocyte apoptosis [52]. In terms of ASICs, Kolker et al [53]. reported that ASIC3 exists in synoviocytes and chondrocytes, and acts as a pH sensor and modulator of hyaluronan expression in response to acidosis induced by acute inflammation. Yuan et al [54]. also reported that administration of amiloride, a non-specific blocker of ASICs, inhibited histological cartilage damage in rat arthritis model. In their latest report *in vitro*, ASICs blocker inhibited acid-induced apoptosis of chondrocytes by increasing anti-apoptotic ability and downregulation of pro-apoptotic factors *via* a mitochondrial-mediated pathway [52]. In summary, current evidence suggests that protons induced by joint inflammation cause cartilage damage through ASICs in chondrocytes, and this theory supports our interesting results of chondroprotection by ASIC3 selective blocker. In this study, APETx2 was effective only in the early phase because it worked against initial inflammation before the severe cartilage damage.

The present study has some limitations. Firstly, knee histology and DRG immunohistochemical staining were only evaluated at 14 days after MIA injection. That is because MIA induced OA is established at Day14 [28]. Time-dependent changes or long term results cannot be discussed. Secondly, MIA-induced OA is a chemically-induced model. Other mechanically-induced OA models may exhibit different pain related characteristics. Thirdly, only one protocol, the same frequency and the same interval of APETx2 administration, was used. The results, especially in weight distribution and knee histology, may change depending on the dose or manner of administration. Lastly, although chondroprotection by APETx2 in OA is an interesting preliminary result, the underlying mechanism is largely unknown. Future research to clarify the molecular mechanism is required.

## Conclusion

In conclusion, local ASIC3 is a pain modulator expressed in joint afferents, which is strongly correlated with weight-bearing pain and secondary hyperalgesia in MIA-induced OA model. APETx2, a selective ASIC3 blocker, inhibited ASIC3 upregulation in knee joint afferents regardless of the time-point of administration. In addition, early administration of APETx2 prevented cartilage damage. APETx2 is a novel, promising drug for OA by relieving pain and inhibiting disease progression.

## Competing interests

The authors have no competing interests to declare in regard to the manuscript entitled: *Local ASIC3 modulates pain and disease progression in a rat model of osteoarthritis.*

## Authors’ contributions

MI was involved in the conception, planning and designing this study, the acquisition of data, analysis and interpretation of data, and writing the manuscript. MI was involved in planning and designing this study, analysis and interpretation of data, and critical revision of the manuscript for important intellectual content. QJ participated in the acquisition of data, analysis and interpretation of data. TT was involved in planning this study and drafting the manuscript. All authors gave final approval of the manuscript.
